# IL-8 and MCP Gene Expression and Production by LPS-Stimulated Human Corneal Stromal Cells

**DOI:** 10.1155/2012/714704

**Published:** 2012-03-18

**Authors:** Roni M. Shtein, Susan G. Elner, Zong-Mei Bian, Victor M. Elner

**Affiliations:** ^1^Department of Ophthalmology and Visual Sciences, University of Michigan Kellogg Eye Center, 1000 Wall Street, Ann Arbor, MI 48105, USA; ^2^Department of Pathology, University of Michigan Kellogg Eye Center, 1000 Wall Street, Ann Arbor, MI 48105, USA

## Abstract

*Purpose*. To determine time course of effect of lipopolysaccharide (LPS) on production of interleukin-8 (IL-8) and monocyte chemotactic protein (MCP) by cultured human corneal stromal cells. *Methods*. Human corneal stromal cells were harvested from donor corneal specimens, and fourth to sixth passaged cells were used. Cell cultures were stimulated with LPS for 2, 4, 8, and 24 hours. Northern blot analysis of IL-8 and MCP gene expression and ELISA for IL-8 and MCP secretion were performed. ELISA results were analyzed for statistical significance using two-tailed Student's *t*-test. *Results*. Northern blot analysis demonstrated significantly increased IL-8 and MCP gene expression after 4 and 8 hours of exposure to LPS. ELISA for secreted IL-8 and MCP demonstrated statistically significant increases (*P* < 0.05) after corneal stromal cell stimulation with LPS. *Conclusions*. This paper suggests that human corneal stromal cells may participate in corneal inflammation by secreting potent leukocyte chemotactic and activating proteins in a time-dependent manner when exposed to LPS.

## 1. Introduction

Leukocytic infiltration of the cornea is a common and important pathologic process observed in infection, autoimmune diseases, allograft rejection, and surgical and nonsurgical trauma. Soluble chemotaxins, such as interleukin-8 (IL-8) and monocyte chemotactic peptide (MCP), both appear to be essential to leukocyte recruitment, accumulation, and activation at sites of inflammation. IL-8 and MCP are distinct polypeptides that directly mediate leukocyte chemotaxis *in vitro* and *in vivo* and may be secreted by tissue-based cells exposed to inflammatory cytokines [1, 2]. Both IL-8 and MCP have been shown to be produced by human corneal tissue in response to inflammatory stimuli [3, 4].

Since the cornea is normally avascular, identification of chemotaxins that elicit corneal leukocyte infiltration may be pertinent to understanding pathogenetic mechanisms regulating corneal inflammation and immunity. Lipopolysaccharide (LPS) is a component of gram-negative bacteria cell membrane that is known to induce the innate immune response. The importance of corneal leukocytic infiltration in disease has prompted prior investigations of corneal-derived chemotactic factors, including lipopolysaccharide (LPS) [5–8], but the specific time- and dose-dependent properties of IL-8 and MCP in response to LPS have not previously been identified.

 In this study, we evaluated cultured human corneal stromal cells for the production of IL-8 and MCP using northern blot analysis to assess gene expression and enzyme-linked immunoabsorbent assays (ELISA) to measure corneal stromal cell secretion of IL-8 and MCP product in response to stimulation by LPS. 

## 2. Materials and Methods

### 2.1. Human Corneal Stromal Cell Cultures

Human donor corneal specimens of good quality, but unsuitable for transplantation, were obtained from the Michigan Eye Bank and Transplantation Center or the Illinois Eye Bank. Within 24 hours of death, corneal stromal cells were harvested by trimming all limbal tissue, mechanically removing the epithelium and endothelial cells, and establishing the corneal stromal cells in Dulbecco's modified essential medium containing 15% fetal bovine serum (FBS). Fourth to sixth passaged cells were used for all assays. Before stimulation with cytokines, the cell cultures were rinsed with fresh, serum-free medium.

### 2.2. Human Corneal Stromal Cell Stimulation with Cytokines

Assayed stromal cells were either left unstimulated or treated with lipopolysaccharide (LPS; Escherichia coli 0111:B4, Sigma Chemical Co., St. Louis, MO) for 2, 4, 8, and 24 hours. Cultured cells were also exposed to LPS at specified concentrations (1, 10, 100, 1000, 10,000 ng/mL) for 8 hours. After experimental incubations, culture media were collected and stored at −70°C until ELISA assays for IL-8 or MCP were performed and the cell monolayers were extracted for IL-8 and MCP mRNA analysis.

### 2.3. Northern Blot Analysis of Human Corneal Stromal Cell IL-8 and MCP mRNA

Human corneal stromal cell monolayers were solubilized in 25 mM TRIS containing 4.2 M guanidine isothiocyanate, 9.5% Sarkosyl, and 0.1 M *β*-mercaptoethanol. Total corneal stromal cell RNA was extracted, and Northern blots prepared by extracting RNA, which was separated by electrophoresis, transferred to nitrocellulose and hybridized with either a ^32^P-5′-end-labeled 30 nucleotide probe complementary to either nucleotides 262 to 291 of the published cDNA sequence for IL-8 or to nucleotides 256 to 285 of the published cDNA sequence for MCP. The blots were washed and autoradiographed. Equivalent amounts of RNA in each Northern blot was assessed by monitoring equivalence of 28s and 18s rRNA signals.

### 2.4. IL-8 and MCP ELISA Assays of Human Corneal Stromal Cell Supernatants

Immunoreactive IL-8 or MCP was measured in corneal stromal cell supernatants using a modification of a double ligand ELISA method. Briefly, 96-well microtiter plates were coated with either rabbit anti-IL-8 or anti-MCP antibody. Sequential incubations with biotinylated rabbit anti-IL-8 (1 : 2000) or anti-MCP then performed and chromogen substrate was added. The plates were incubated to the desired extinction and the reaction terminated. Plates were read in an ELISA reader and calibrated using 1/2 log dilution standards of rIL-8 or rMCP concentrations ranging from 1 pg to 1000 ng/well. Under each condition, the ELISA was performed 1 mL of media covering 500,000 cells. This ELISA method consistently detected IL-8 or MCP concentrations greater than 10 pg/mL in a linear fashion.

### 2.5. Statistical Analysis

Individual experiments were performed 4 times on 4 different corneal stromal cell lines. All data were expressed as means ± standard error of the means. The various assay conditions were compared using Student's *t*-test and *P*-values less than 0.05 were considered to be statistically significant.

## 3. Results and Discussion

**Figure 1:**
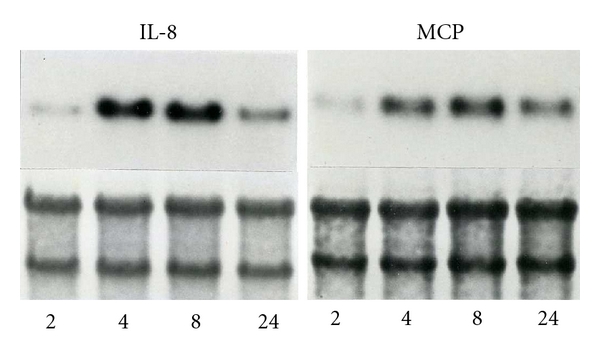
Time-dependent human corneal stromal cell mRNA expression of IL-8 and MCP detected by Northern blot analysis following lipopolysaccharide (LPS) stimulation of 2, 4, 8, and 24 hours.

Northern blot analysis was performed to assess mRNA expression of IL-8 and MCP by human corneal stromal cells in response to varying exposure times (2, 4, 8, and 24 hours) to LPS at a concentration of 1000 ng/mL. mRNA expression of both IL-8 and MCP was demonstrated to be minimal after 2 hours of exposure to LPS, but then increased substantially after 4 to 8 hours of exposure to LPS (Figure 1). mRNA expression of both IL-8 and MCP was decreased after 24 hours of LPS exposure, with a greater decrease seen in IL-8 than MCP expression (Figure 1). No induction or mock induction controls were used in this analysis.

**Figure 2:**
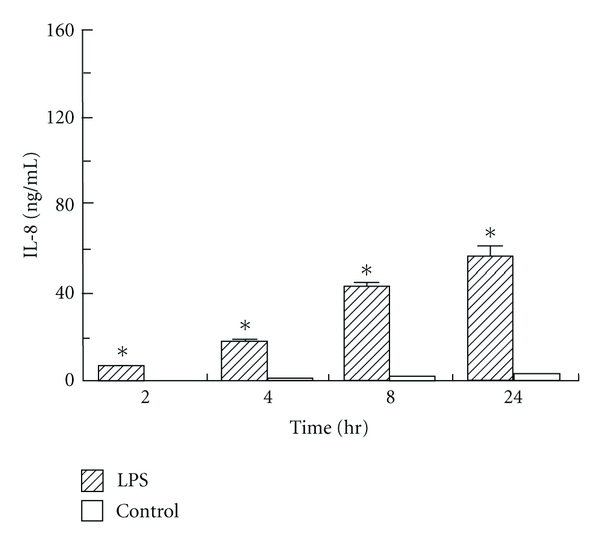
Time-dependent human corneal stromal cell secretion of IL-8 detected by ELISA following lipopolysaccharide (LPS) stimulation of 2, 4, 8, and 24 hours. *P < 0.05 compared to unstimulated control.

**Figure 3:**
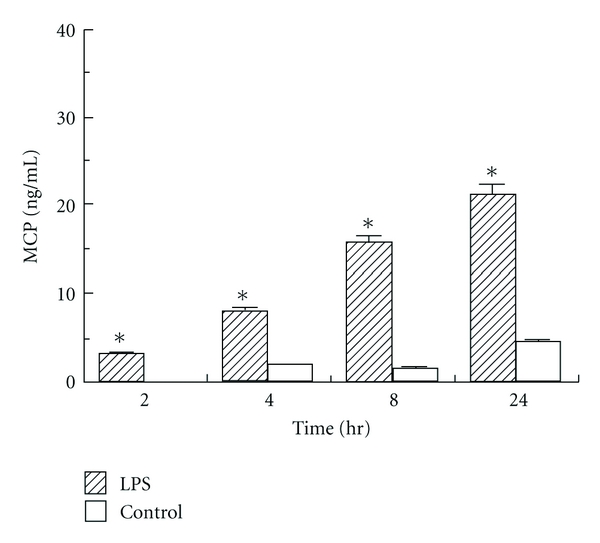
Time-dependent human corneal stromal cell secretion of MCP detected by ELISA following lipopolysaccharide (LPS) stimulation of 2, 4, 8, and 24 hours. *P < 0.05 compared to unstimulated control.

ELISA was performed to assess the expression of IL-8 and MCP by human corneal stromal cells after exposure to LPS (1000 ng/mL) for 2, 4, 8, and 24 hours. A low level of constitutive IL-8 and MCP was consistently determined on unstimulated control cells. A substantial increase was seen relative to unstimulated control with stimulation of LPS as soon as 2 hours after exposure, and at all measured time points (*P* < 0.05) (Figures 2 and 3). Expression of both IL-8 and MCP-1 increased progressively with length of time of exposure to LPS (*P* < 0.05) (Figures 2 and 3).

**Figure 4:**
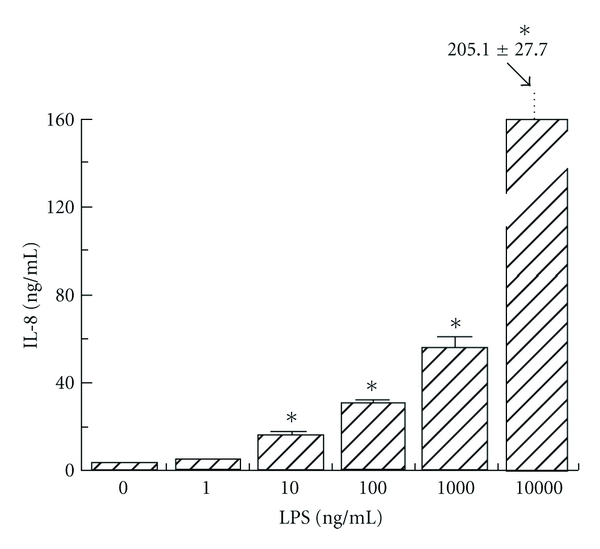
Dose-dependent human corneal stromal cell secretion of IL-8 detected by ELISA following lipopolysaccharide (LPS) stimulation at 1, 10, 100, 1,000, and 10,000 ng/mL concentrations. *P < 0.05 compared to unstimulated control.

**Figure 5:**
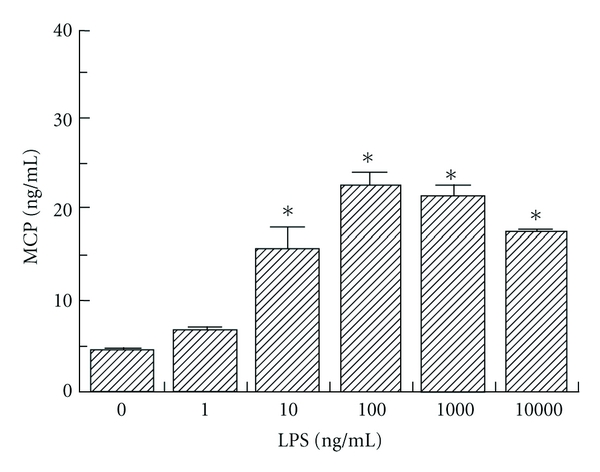
Dose-dependent human corneal stromal cell secretion of MCP detected by ELISA following lipopolysaccharide (LPS) stimulation at 1, 10, 100, 1,000, and 10,000 ng/mL concentrations. *P < 0.05 compared to unstimulated control.

To determine the effect of LPS concentration on human corneal stromal cell IL-8 and MCP expression, cells were incubated for 8 hours with different concentrations of LPS (1, 10, 100, 1,000, and 10,000 ng/mL). ELISA was used to compare the IL-8 and MCP expression at the different concentrations of LPS. Increasing concentrations of LPS led to progressive increases in IL-8 expression which were statistically significant (*P* < 0.05) (Figure 4). There was also increased MCP expression with increasing concentrations of LPS, but MCP expression reached a plateau at LPS concentrations greater than 100 ng/mL (Figure 5). MCP expression was statistically significantly increased from baseline at all concentrations greater than 1 ng/mL (*P* < 0.05).

## 4. Conclusions

Circulating leukocytes infiltrate tissue at sites of inflammation by binding to cellular adherence molecules expressed by tissue-based cells and migrating along gradients of specific chemotactic factors. Although the precise stimuli initiating inflammation vary, they appear to trigger common pathogenetic cascades leading to leukocyte elicitation and activation. LPS is an important mediator of inflammation that has been shown to induce de novo synthesis of specific leukocyte chemotaxins, such as IL-8 and MCP, by tissue-based cells.

 Our results show that IL-8 and MCP are chemotaxins that are significantly induced in corneal stromal cells by LPS stimulation. Our findings suggest that LPS stimulation of steady-state corneal stromal cell IL-8 and MCP mRNA is prompt and is subsequently followed by secretion of significant levels of these cytokines human corneal stromal cells.

This induction is time dependent, with exposures of 4 to 8 hours inducing the highest increase in mRNA expression seen in our assays. There is then a decrease in mRNA expression at 24 hours, possibly indicating a saturation effect of this pathway of acute inflammation. This decrease was more marked with IL-8 mRNA expression than MCP, likely associated with the role of MCP in more chronic inflammation.

Our results show that both IL-8 and MCP protein expression continues to increase over the first 24 hours of exposure to LPS. It would be interesting to proceed with a longer-term study to evaluate the time dependency of the protein expression of these chemokines. Our study also evaluates the effect of concentration of LPS on protein expression of IL-8 and MCP by corneal stromal cells. IL-8 expression increased dramatically in response to increasing concentrations of LPS. On the other hand, MCP expression reached a maximum at an LPS concentration of 100 ng/mL, and then seemed to plateau or even show slight decreases in expression at higher concentrations of LPS. This unusual dose response at higher concentrations requires further study.

Previous studies have shown that LPS from several different bacterial species induces increased IL-8 and MCP mRNA by quantitative RT-PCR and increased protein expression by ELISA [5]. It has also been shown that increased IL-8 and MCP mRNA and protein expression in corneal fibroblasts is potentiated by LPS-binding protein and soluble CD14 [6]. To our knowledge, this is the first study to show the time-course and dose dependence of the induction of IL-8 and MCP in human corneal stromal cells after exposure to LPS.

Once secreted, corneal-derived IL-8 and MCP may elicit their inflammatory effects by binding specific receptors on neutrophils, lymphocytes, and monocytes, leading to leukocyte accumulation and activation at the site of inflammation [3, 4]. The elaboration of IL-8 and MCP by tissue-based cells may be an especially important mechanism directing leukocytes to migrate over long distances through tissue in which blood vessels containing circulating leukocytes are absent, such as the normally avascular cornea. The induction of IL-8 and MCP is one of many events that occur in response to LPS. Thus, corneal stromal keratocytes may produce soluble, diffusible chemotaxins, including IL-8 and MCP, that may be important in the inflammatory cascade response to LPS stimulation.
